# The role of galectins in modulating the tumor microenvironment and driving metastasis in skin cancers

**DOI:** 10.1186/s40659-026-00695-w

**Published:** 2026-05-13

**Authors:** Mohsen Dabagh, Alina Ciceu, Cornel Balta, Maria Consiglia Trotta, Bartolo Ferraro, Giovanbattista D’Amico, Anca Hermenean, Vlad Mihail Voiculescu

**Affiliations:** 1 Multidisciplinary Doctoral School, Vasile Goldis Western University of Arad, Arad, Romania; 2 Institute of Life Sciences, Vasile Goldis Western University of Arad, Arad, Romania; 3 Department of Experimental Medicine, University of Campania Luigi Vanvitelli , Caserta, Italy; 4 Institute of Cardiovascular Physiology and Pathophysiology, Biomedical Center, Ludwig- Maximilian-University Munich, Munich, Germany; 5 Medizinische Klinik und Poliklinik I, Klinikum der Universität, Ludwig- Maximilians-University Munich, Munich, Germany; 6 School of Geriatrics, University of Studies of L’Aquila, 67010 L’Aquila, Italy; 7 Department of Histology, Faculty of Medicine, Vasile Goldis Western University of Arad, Arad, Romania; 8 Department of Dermatology, Carol-Davila University of Medicine and Pharmacy, Bucharest, Romania; 9 Department of Dermatology, Elias University Emergency Hospital, Bucharest, Romania

**Keywords:** Skin cancer, Galectins, Tumor microenvironment, Immune evasion, Metastasis, Therapy resistance, Biomarkers

## Abstract

Galectins, a family of β-galactoside–binding lectins, are key regulators of tumor progression, immune evasion, and therapy resistance in skin cancers. Among them, Galectin-1, Galectin-3, and Galectin-9 have been most extensively investigated for their multifaceted roles in shaping the tumor microenvironment. These proteins influence cancer cell proliferation, apoptosis, angiogenesis, and immune cell responses through both intracellular and extracellular mechanisms. Galectin-1 and Galectin-3 modulate immune checkpoint pathways and cancer-associated fibroblast activation, whereas Galectin-9 interacts with TIM-3 to induce T-cell exhaustion. Other family members, including Galectin-7 and Galectin-8, display context-dependent functions that may either promote or inhibit tumor development. Recent preclinical and early clinical trials targeting galectins, such as the Gal-3 inhibitors belapectin and GB1211, highlight their translational potential as adjuncts to immunotherapy. This review summarizes current knowledge on galectin expression and function in melanoma and non-melanoma skin cancers, emphasizing their contribution to immune escape and therapeutic resistance, as well as challenges and perspectives for galectin-targeted interventions.

## Introduction

Galectins are a family of β-galactoside–binding lectins widely expressed in mammalian tissues, where they participate in cell–cell and cell–matrix interactions, immune modulation, and tissue homeostasis [1]. Structurally, galectins are classified into three groups—prototype (e.g., Galectin-1 and − 7), tandem-repeat (e.g., Galectin-8 and − 9), and chimera-type (Galectin-3)—based on the organization of their carbohydrate recognition domains (CRDs) [2–4]. Despite lacking a classical signal peptide, galectins are secreted through non-conventional pathways, including exosome release and autophagy-related vesicular transport, enabling them to function both intracellularly and extracellularly [5, 6].

Within the tumor microenvironment (TME), galectins regulate multiple processes that sustain cancer progression. They influence tumor growth, angiogenesis, epithelial–mesenchymal transition (EMT), and immune escape by interacting with glycoconjugates on tumor and immune cell surfaces [7, 8]. Galectin-1 and Galectin-3, in particular, contribute to immune evasion through modulation of T-cell apoptosis, dendritic cell tolerance, and PD-1/PD-L1 stabilization [9, 10]. Galectin-9 engages TIM-3 to drive T-cell exhaustion and promote immune suppression [11, 12]. These mechanisms collectively establish an immunosuppressive milieu that promotes tumor survival.

Skin cancers, including melanoma and non-melanoma types such as squamous cell carcinoma (SCC) and basal cell carcinoma (BCC), represent distinct settings in which galectins exert diverse and context-dependent roles. Recent studies highlight their dual activity in tumor and stromal compartments, influencing immune evasion, epithelial–mesenchymal transition, and metastatic dissemination [3, 13]. Beyond their extracellular glycan-binding-dependent functions, Galectin-3, Galectin-9 and Galectin 8 also play a role in the cytosolic compartment maintaining cellular integrity through endolysosomal damage repair and autophagy regulation [10, 14, 15]. Other members, including Galectin-7 and Galectin-8, have emerged as modulators of keratinocyte differentiation, cutaneous inflammation, and cancer cell migration, underscoring the multifaceted contribution of galectins to skin cancer biology [8, 16].

Given their pleiotropic roles in tumor biology, galectins have emerged as promising therapeutic targets. Preclinical and early clinical studies of galectin inhibitors—such as the Galectin-3 antagonists belapectin (GR-MD-02) and GB1211—demonstrate potential to improve the efficacy of immune checkpoint inhibitors by modulating the tumor microenvironment [17, 18]. Nevertheless, the functional redundancy and context-dependent effects of different galectin family members necessitate a more refined understanding of their dual roles in tumor promotion and suppression [3].

This review integrates current evidence on galectin expression and function in melanoma and non-melanoma skin cancers, with a focus on their contributions to immune modulation, tumor progression, and therapeutic resistance, while outlining emerging strategies for galectin-targeted interventions.

## General features of the tumor microenvironment in skin cancers

The TME is a highly dynamic and complex network of cellular and non-cellular components that play a crucial role in the initiation, progression, and therapeutic response of skin cancers. Unlike intrinsic oncogenic mutations within tumor cells, the TME consists of stromal and immune cells, ECM components, and soluble factors that collectively shape tumor behavior. In skin cancers, the unique influence of environmental factors, such as ultraviolet (UV) radiation, further modulates the TME, leading to distinct molecular and cellular adaptations [19]. Understanding the composition and function of the TME in skin cancers is essential for identifying novel therapeutic targets and overcoming resistance mechanisms.

### Main components of the tumor microenvironment

#### Stromal cells

The stromal compartment of the TME comprises fibroblasts, cancer-associated fibroblasts (CAFs), and endothelial cells, all of which contribute to tumor progression through extracellular matrix remodeling, angiogenesis, and the secretion of growth factors [20]. Fibroblasts in normal skin maintain tissue homeostasis, but upon transformation into CAFs, they promote tumor growth by secreting pro-tumorigenic cytokines, such as transforming growth factor-beta (TGF-β) and fibroblast growth factors (FGFs) [21]. Endothelial cells, which form the tumor vasculature, facilitate angiogenesis—a key process that supplies oxygen and nutrients to growing tumors [22]. The interplay between these stromal elements fosters a supportive niche for tumor cell survival and metastasis.

#### Immune cells

The immune landscape of the skin cancer TME is highly diverse and plays a dual role, contributing to both anti-tumor immunity and immune evasion. Among the most influential immune cells are tumor-associated macrophages (TAMs), which exist along a spectrum between pro-inflammatory (M1) and immunosuppressive (M2) phenotypes [23]. In melanoma and non-melanoma skin cancers, M2-polarized TAMs secrete immunosuppressive cytokines such as IL-10 and TGF-β, while also promoting angiogenesis, thereby facilitating tumor progression [24].

Another key player in the immune TME is the T cell population. Cytotoxic T lymphocytes (CTLs) are crucial for recognizing and eliminating tumor cells. However, in an immunosuppressive TME, the presence of regulatory T cells (Tregs) and exhausted CD8 + T cells dominates, ultimately reducing effective anti-tumor immunity [25]. Additionally, dendritic cells (DCs), which are professional antigen-presenting cells (APCs) responsible for activating T cells, often exhibit impaired functionality in the tumor milieu, leading to ineffective immune responses [26].

Furthermore, tumor-associated neutrophils (TANs) exert context-dependent effects, either promoting or suppressing tumor growth. In skin cancers, pro-tumor TANs release reactive oxygen species (ROS) and form neutrophil extracellular traps (NETs), which contribute to tumor invasion and progression [27]. Ultimately, the immune balance within the TME determines whether skin cancers successfully evade immune surveillance or become susceptible to immunotherapeutic interventions, such as immune checkpoint inhibitors.

#### Extracellular matrix (ECM)

The ECM serves as a structural scaffold that provides mechanical support and modulates biochemical signaling pathways involved in tumor progression. ECM components, including collagens, laminins, and proteoglycans, undergo significant remodeling in skin cancers, influencing cell adhesion, migration, and proliferation [28]. Enzymes such as matrix metalloproteinases (MMPs) degrade ECM proteins, facilitating tumor invasion and metastasis. Additionally, ECM stiffness, often increased in aggressive tumors, alters mechanotransduction pathways that enhance tumor cell survival [29].

**Fig. 1:**
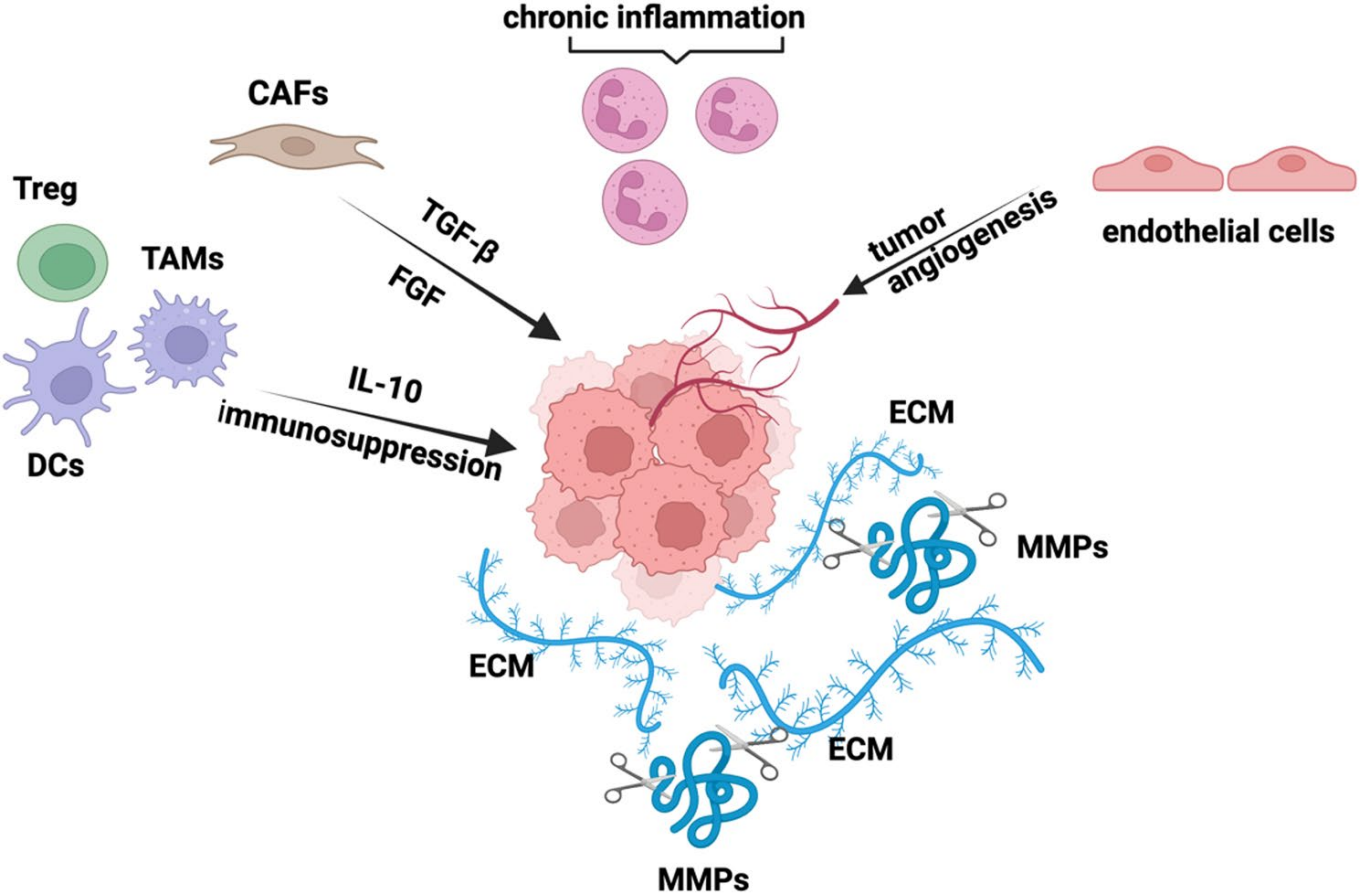
Key components of the tumor microenvironments which contribute to tumor progression, immune evasion, and metastatic dissemination; CAFs secrete TGF-β and FGF, promoting tumor growth and stromal remodeling. TAMs and Tregs contribute to immunosuppression, particularly through IL-10 secretion, allowing tumors to evade immune detection. DCs, while typically involved in antigen presentation, are often impaired within the TME, further dampening anti-tumor immunity. Chronic inflammation, marked by the presence of neutrophils, supports tumor development and facilitates a pro-tumorigenic environment. Endothelial cells play a crucial role in tumor angiogenesis, forming new blood vessels that supply nutrients and oxygen to the tumor, further supporting its growth and metastatic potential. ECM components, including collagen fibers, provide structural integrity but are actively remodeled by MMPs, which degrade ECM proteins to facilitate tumor invasion and metastasis. Created with Biorender.com

Figure 1 illustrates the complex interactions within the TME, highlighting the roles of immune cells, stromal components, ECM remodeling, and angiogenesis in tumor progression.

### Unique aspects of the tumor microenvironment in skin cancers

TME in skin cancers exhibits distinct characteristics shaped by UV radiation exposure, chronic inflammation, and the unique biology of melanocytes in melanoma [30]. These factors contribute to tumor progression, immune evasion, and therapy resistance, making skin cancers particularly challenging to treat.

A defining feature of skin cancer TME is UV radiation-induced damage, which promotes DNA mutations, oxidative stress, and immunosuppression [25]. UV exposure leads to characteristic genetic alterations, such as BRAF mutations in melanoma and TP53 mutations in SCC, facilitating tumor initiation [31, 32]. Additionally, it disrupts immune surveillance by impairing DCs function, reducing antigen presentation, and promoting Treg cell expansion, which suppresses anti-tumor immune responses [33–35].

Chronic inflammation is another hallmark of the skin cancer TME, particularly in SCC, which often arises in areas of persistent injury [36–38]. TAMs and neutrophils contribute to this inflammatory environment by secreting immunosuppressive cytokines (IL-10, TGF-β) and ROS, which promote DNA damage and tumor progression [39, 40]. Furthermore, CAFs remodel ECM, enhancing tumor invasion through the secretion of matrix metalloproteinases (MMPs) [41, 42].

In melanoma, the microenvironment is further influenced by the intrinsic plasticity of melanocytes, which allows tumor cells to rapidly adapt to environmental pressures [43]. Melanoma cells evade immune detection by upregulating PD-L1, suppressing cytotoxic T cell activity [44], and demonstrating metabolic flexibility, shifting between glycolysis and oxidative phosphorylation to sustain growth [45]. This high adaptability makes melanoma particularly resistant to conventional therapies.

Together, these factors create a highly dynamic and immunosuppressive TME, allowing skin cancers to thrive despite immune surveillance and therapeutic interventions. Targeting immune checkpoints, inflammatory mediators, and ECM remodeling processes represents a promising strategy to improve treatment outcomes in melanoma, SCC, and BCC.

Table 1 compares key characteristics of melanoma, SCC and BCC, highlighting their cellular origins, interactions with the immune system, and mechanisms of tumor progression.

**Table 1:** Comparative analysis of tumor microenvironment in skin cancers

Feature	Melanoma	Squamous cell carcinoma (SCC)	Basal cell carcinoma (BCC)
Cell of origin	Melanocytes (pigment-producing cells)	Keratinocytes (epidermal skin cells)	Keratinocytes (basal layer of epidermis)
UV radiation influence	High – UV-induced DNA mutations (e.g., BRAF, NRAS, TP53) [46, 47]	High – Chronic sun exposure is a key factor [48, 49]	Moderate – Less linked to UV than SCC and melanoma [50]
Inflammation & Immune response	Immunogenic; high T-cell infiltration but strong immune evasion mechanisms [51]	Chronic inflammation promotes carcinogenesis [52]	Low immunogenicity; often evades immune detection [53]
Angiogenesis & Hypoxia	Highly vascularized; VEGF-driven angiogenesis supports metastasis [54]	Moderate angiogenesis [55]	Less vascularized, grows slowly [55]
Tumor-associated fibroblasts (CAFs)	Highly active; contribute to metastasis and drug resistance [56]	Present and contribute to extracellular matrix remodeling [57]	Present but less active than in melanoma and SCC [58, 59]
Extracellular matrix (ECM) Remodeling	Extensive ECM degradation (MMPs, integrins) promotes invasion [60–62]	High ECM remodeling for local invasion [63]	Limited ECM remodeling; tumor remains localized [64]
Key immune cells in TME	Tumor-associated macrophages (TAMs), regulatory T cells (Tregs), exhausted CD8 + T cells [43, 65, 66]	Tumor-associated neutrophils (TANs), macrophages, T cells [67, 68]	Fewer immune infiltrates, mostly innate immune cells [53]

## Expression of galectins in skin cancers

Galectins, a family of β-galactoside-binding proteins, are key regulators of tumor progression, immune modulation, and metastatic dissemination in skin cancers [69]. Their expression varies across melanoma, SCC and BCC, influencing tumor cell adhesion, angiogenesis, immune evasion, and ECM remodeling. Among them, galectin-1 (Gal-1) and galectin-3 (Gal-3) have been extensively studied due to their significant roles in tumor aggressiveness and therapy resistance [13, 70].

Galectins are synthesized in the cytosol and, due to the absence of a signal peptide, they bypass the conventional ER/Golgi secretory pathway. Instead, they are exported through non-classical mechanisms. For example, Galectin-3 has been described to be secreted via exosomes [5], while Galectin-9 and other family members employ alternative unconventional secretion routes, including autophagy-independent vesicular transport [6, 71]. These secretion mechanisms are essential for their extracellular localization, where they modulate cell–cell and cell–matrix interactions in the tumor microenvironment.

Beyond their extracellular activities, galectins also exert important cytosolic functions. Galectin-3 and Galectin-9, in particular, act as intracellular danger sensors of endolysosomal damage. Upon lysosomal injury, these galectins rapidly accumulate on damaged vesicles, where they orchestrate selective autophagy (“lysophagy”) or lysosomal repair, preventing uncontrolled release of proteases into the cytosol and ensuring cellular survival under stress conditions [10, 14]. This dual functionality highlights the importance of distinguishing between intracellular and extracellular galectin activities in the context of cancer progression. In the intracellular compartment, Gal-3 and Gal-9, together with Gal-8 configure a system of sensors of endomembrane damage, which recognizes injured lysosomes and plays complementary roles in promoting their repair or selective autophagy (lysophagy), thereby preventing uncontrolled leakage of proteases and ensuring cell survival under stress conditions [10, 14, 15].

Glycan structures recognized by galectins are dynamically remodeled in cancer and other pathological conditions. Of special interest are branched N-glycans, which enhance galectin binding affinity and lattice formation, thereby influencing adhesion, signaling, and tumor progression [72–76].

In addition to Gal-1, Gal-3, and Gal-9, other members of the galectin family have also been implicated in skin cancers (Table 2). Galectin-7, initially identified as a p53-induced gene in keratinocytes, has been linked to both pro-apoptotic and pro-metastatic roles, underscoring its context-dependent function in squamous cell carcinoma and melanoma [84, 85]. Galectin-8, a tandem-repeat galectin, can modulate integrin signaling and cell adhesion, thereby influencing tumor invasion and angiogenesis, although its role in cutaneous malignancies remains less well-defined [82]. Including these galectins provides a more comprehensive perspective on the diversity of galectin functions in skin cancer biology.

**Table 2:** Summary of galectin family members implicated in skin cancers

Galectin	Expression in skin cancer	Reported functions	References
Gal-1	Melanoma, SCC	Immune evasion, angiogenesis, CAF activation	[7, 77]
Gal-3	Melanoma, BCC, SCC	Immune evasion, apoptosis resistance, PD-L1 stabilization, adhesion/migration	[78, 79]
Gal-7	SCC, melanoma	Pro-apoptotic in keratinocytes; pro-metastatic in SCC; context-dependent	[80, 81]
Gal-8	Limited data in skin cancers	Integrin modulation, adhesion, angiogenesis, immune regulation	[82]
Gal-9	Melanoma, SCC	TIM-3 binding, T cell exhaustion, dual role in prognosis	[11, 83]

The table outlines their reported expression patterns, functional roles, and relevant references. Galectin-1, Galectin-3, and Galectin-9 remain the best-characterized, while evidence for Galectin-7 and Galectin-8 suggests additional, context-dependent contributions to tumor biology and therapy resistance

### Profiles of galectin expression in skin cancers

Gal-1 and Gal-3 are highly expressed in melanoma and SCC, where they contribute to immune suppression and metastatic potential. In melanoma, Gal-1 promotes T-cell apoptosis, Treg expansion, and vascular endothelial growth factor (VEGF)-mediated angiogenesis, thereby supporting tumor progression and immune evasion [81, 86]. Similarly, in SCC, Gal-1 facilitates extracellular matrix remodeling and fibroblast activation, enhancing tumor invasion and local progression [87]. Gal-3, on the other hand, plays a crucial role in tumor adhesion, migration, and apoptosis resistance by interacting with integrins and ECM components, particularly in melanoma and advanced SCC [88, 89]. In contrast, BCC exhibits lower expression of Gal-3 [90]. Although less frequently investigated, basal cell carcinoma (BCC) also exhibits distinct galectin expression patterns. Galectin-3 levels are typically reduced in BCC compared with adjacent normal skin, while Galectin-7 dysregulation has been associated with altered keratinocyte differentiation and local inflammatory responses [8, 16, 90, 91].

Other galectins also contribute to skin cancer pathogenesis. Galectin-9 (Gal-9), for instance, exerts dual effects in melanoma, acting as both a pro-apoptotic factor and an immune suppressor by binding to TIM-3 receptors on T cells, leading to T-cell exhaustion and tumor immune escape [92, 93]. Meanwhile, Galectin-7 (Gal-7), typically involved in keratinocyte homeostasis, has been found to be upregulated in SCC, associated with activation of ERK and JNK signaling and the induction of MMP-2 and MMP-9 and tumor invasion [81]. Additionally, a study found that galectin-8 expression is significantly reduced in dysplastic and malignant squamous epithelium of the head and neck, potentially contributing to malignant transformation [94].

## Functional roles of galectins in tumor microenvironment

It is important to differentiate between intracellular and extracellular functions of galectins when analyzing their impact on the tumor microenvironment. Overexpression or knockdown of galectins can influence both intracellular pathways—such as endolysosomal repair, autophagy regulation, and mTOR signaling—and extracellular signaling mechanisms following their secretion [72, 73]. Notably, extracellular galectins can modulate receptor signaling; for example, Galectin-8 has been shown to induce trans-activation of the epidermal growth factor receptor (EGFR) from the extracellular environment [95]. Glycan structures recognized by galectins are dynamically remodeled in cancer and other pathological conditions. All N-glycans are inherently branched; however, variations in their degree of branching critically influence galectin-binding affinity [74–76]. In addition to branching of complex-type N-glycans, other structural features such as repeated N-acetyllactosamine (LacNAc) motifs and post-glycosylation modifications—including fucosylation, sialylation, and sulfation of galactose residues—play key roles in determining galectin specificity and binding strength [72–74, 76, 96]. Furthermore, alterations in the glycan landscape under inflammatory conditions, such as the loss of sialic acid mediated by neuraminidases, can shift galectin interactions, for example from Galectin-8 to Galectin-3, thereby modulating downstream signaling and cellular responses [97, 98].

The tumor microenvironment of skin cancer is a complex network where stromal cells play a pivotal role in supporting cancer progression [99]. Galectins, particularly Gal-1 and Gal-3, are crucial modulators of stromal cell activity, influencing processes such as fibroblast activation and angiogenesis [100].

CAFs are key players in the remodeling of the ECM, promoting a tumor-friendly niche [101]. Galectins enhance CAF activation by upregulating signaling molecules such as TGF-β and FGF-2, which in turn stimulate ECM deposition and fibrosis [41, 102, 103].

In addition, galectins promote angiogenesis by directly interacting with endothelial cells [104]. For example, Galectin-3 promotes melanoma progression by regulating autotaxin expression through NFAT1, influencing tumor growth, angiogenesis, and metastasis [88]. This neovascularization provides essential nutrients and oxygen to the growing tumor, facilitating its expansion and survival.

Recent insights have revealed that the tumor vasculature exerts functions that extend beyond oxygen and nutrient supply. Endothelial cells actively participate in tumor progression through angiocrine signaling, secreting paracrine factors such as VEGF, FGF2, IL-8, and angiopoietins, which influence cancer cell proliferation, stemness, immune evasion, and therapeutic resistance [105–108]. In skin cancers, such angiocrine cues may cooperate with galectin-mediated pathways to reinforce epithelial–mesenchymal plasticity and immune suppression within the tumor microenvironment.

Galectins contribute significantly to immune evasion mechanisms in skin cancer [109]. These proteins suppress anti-tumor immunity by modulating T cell function, altering macrophage polarization, and impairing DCs activity [69, 110].

T cell suppression occurs through multiple mechanisms. Gal-1 induces apoptosis in activated CD8 + cytotoxic T cells, thereby reducing the immune system’s ability to attack tumor cells [111, 112]. Gal-1 induces apoptosis by activating the AP-1 transcription factor and downregulating Bcl-2 [113]. Gal-3 functions as an immune regulator that directly influences T cell activation, leading to apoptosis and suppression of anti-tumor responses, ultimately promoting tumor growth [114]. This suggests a novel mechanism of tumor immune tolerance, where high levels of Gal-3 inhibit tumor-reactive T cells, contributing to immune evasion and tumor progression.

Macrophages within the TME often exhibit a pro-tumoral M2 phenotype under the influence of galectins [115]. Gal-3, in particular, promotes macrophage polarization towards the M2 state by activating STAT3 and IL-10 signaling [116, 117]. These M2 macrophages contribute to immunosuppression by secreting anti-inflammatory cytokines and promoting tissue remodeling [115, 118].

Dendritic cells, which are crucial for antigen presentation and T cell activation, are also affected by galectins [109, 119]. Gal-1 and Gal-9 impair DC maturation and antigen-presenting capacity, leading to reduced activation of cytotoxic T cells and increased tolerance to tumor antigens [12, 120].

Recent evidence has further elucidated the mechanistic pathways through which galectins modulate immune suppression and epithelial–mesenchymal plasticity in skin cancers. Galectin-3 directly stabilizes PD-L1 on tumor cell surfaces by preventing its ubiquitin-mediated degradation, thereby sustaining T-cell exhaustion and contributing to immune checkpoint inhibitor resistance [17, 121]. Similarly, Galectin-1 promotes epithelial–mesenchymal transition (EMT) via activation of the TGF-β/Smad and PI3K/AKT signaling cascades, upregulating transcription factors such as Snail, Twist, and ZEB1 that drive invasion and metastasis [72, 122]. These intertwined mechanisms underscore the dual intracellular and extracellular actions of galectins in shaping an immunosuppressive and pro-metastatic tumor microenvironment.

**Fig. 2:**
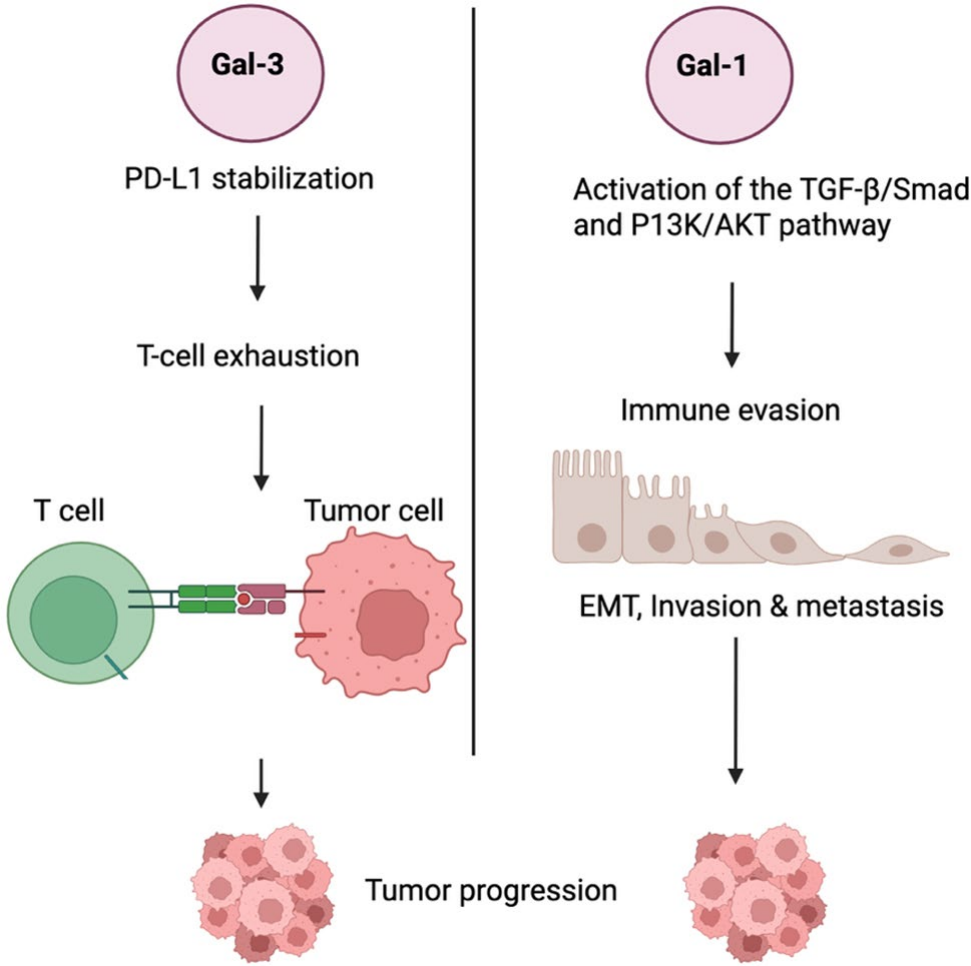
Roles of Galectin-1 and Galectin-3 in cancer progression. Created in Biorender

As illustrated in Fig. 2, Galectin-3 and Galectin-1 regulate complementary yet interconnected mechanisms that sustain tumor immune evasion and promote epithelial–mesenchymal transition (EMT), thereby contributing to skin cancer progression and resistance to immune-based therapies.

## Therapy resistance and galectins in skin cancers

Skin cancers, including melanoma and NMSC such as SCC and BCC, often develop resistance to standard therapies, limiting treatment efficacy and patient survival [123]. Galectins, a family of β-galactoside-binding lectins, play a crucial role in modulating immune evasion, drug resistance, and radiotherapy response, making them key players in therapy resistance mechanisms [124]. Their ability to interact with glycosylated immune checkpoints, regulate survival pathways, and modulate the TME has placed them at the forefront of emerging therapeutic targets [125, 126].

### Immunotherapy resistance: galectins and immune checkpoint suppression

Immunotherapy, particularly immune checkpoint inhibitors (ICIs) targeting PD-1, PD-L1, and CTLA-4, has significantly improved survival in melanoma [127, 128]. However, resistance to ICIs is a growing concern, and galectins have been identified as key modulators of immune escape. As detailed in Sect.“Functional roles of galectins in tumor microenvironment”, Gal-1/3-driven immune evasion and PD-1/PD-L1 stabilization contribute to ICI resistance.

Blocking galectin signaling has been proposed as a strategy to restore immune function in the TME. Gal-3 modulates immune checkpoint inhibition in metastatic malignant melanoma (MM) by sterically blocking the binding of pembrolizumab to PD-1, thereby contributing to immune suppression in the tumor microenvironment and influencing treatment response [129]. In another study, Gal-3 enhances the PD-1/PD-L1 interaction, reducing the efficacy of immune checkpoint inhibitors like pembrolizumab and atezolizumab, while inhibition of Gal-3 with GB1211 restores therapeutic binding and improves anti-tumor immune responses, highlighting its potential as a combinatory target in immunotherapy [121]. Combining galectin inhibitors with ICIs may therefore provide a novel strategy to overcome primary and acquired resistance in skin cancers.

Collectively, these mechanisms contribute to resistance against immune checkpoint blockade and stromal-targeted therapies.

Several galectin-targeting agents have progressed from preclinical studies into clinical testing. Belapectin (GR-MD-02), a galactoarabino-rhamnogalacturonate polysaccharide that selectively inhibits Galectin-3, has been evaluated in phase II trials for liver fibrosis and, more recently, in oncology settings in combination with immune checkpoint inhibitors [17, 130]. Early data suggest that belapectin may improve the efficacy of pembrolizumab in patients with metastatic melanoma and head and neck cancers. Another promising agent is GB1211, a potent oral Galectin-3 inhibitor with improved bioavailability compared to belapectin, currently tested in phase I/II studies for solid tumors [18]. These examples illustrate the translational potential of galectin inhibition and support the rationale for exploring galectin-targeted therapies in skin cancers, either as monotherapy or in combination with existing immunotherapies.

### Chemoresistance: galectins and pro-survival signaling

Despite advances in targeted therapies, chemotherapy remains a mainstay treatment, particularly for metastatic melanoma and SCC. However, chemoresistance significantly reduces treatment success. Galectins contribute to drug resistance by modulating drug efflux mechanisms, pro-survival signaling pathways, and EMT [131].

Gal-3 and Gal-1 are known to upregulate ATP-binding cassette (ABC) transporters such as P-glycoprotein (P-gp) and MRP1, which actively pump chemotherapeutic agents out of cancer cells, reducing intracellular drug accumulation and cytotoxicity [132, 133].

Additionally, galectins activate pro-survival pathways such as PI3K/AKT and MAPK, which protect tumor cells from apoptosis induced by chemotherapeutic agents [134]. Galectin-mediated activation of these pathways is associated with increased expression of anti-apoptotic proteins such as Bcl-2 and Mcl-1, further reducing the efficacy of cytotoxic drugs [135].

Galectins also contribute to chemoresistance by promoting EMT, a process that enhances cancer cell plasticity and invasiveness [72]. Galectin-1 has been shown to induce EMT by upregulating ZEB1, Snail, and Twist, transcription factors that suppress E-cadherin expression, facilitating drug-resistant mesenchymal phenotypes [72, 136]. This is particularly relevant in melanoma, where EMT-like transitions are associated with resistance to BRAF inhibitors (vemurafenib, dabrafenib) and MEK inhibitors (trametinib, cobimetinib) [137, 138].

**Fig. 3:**
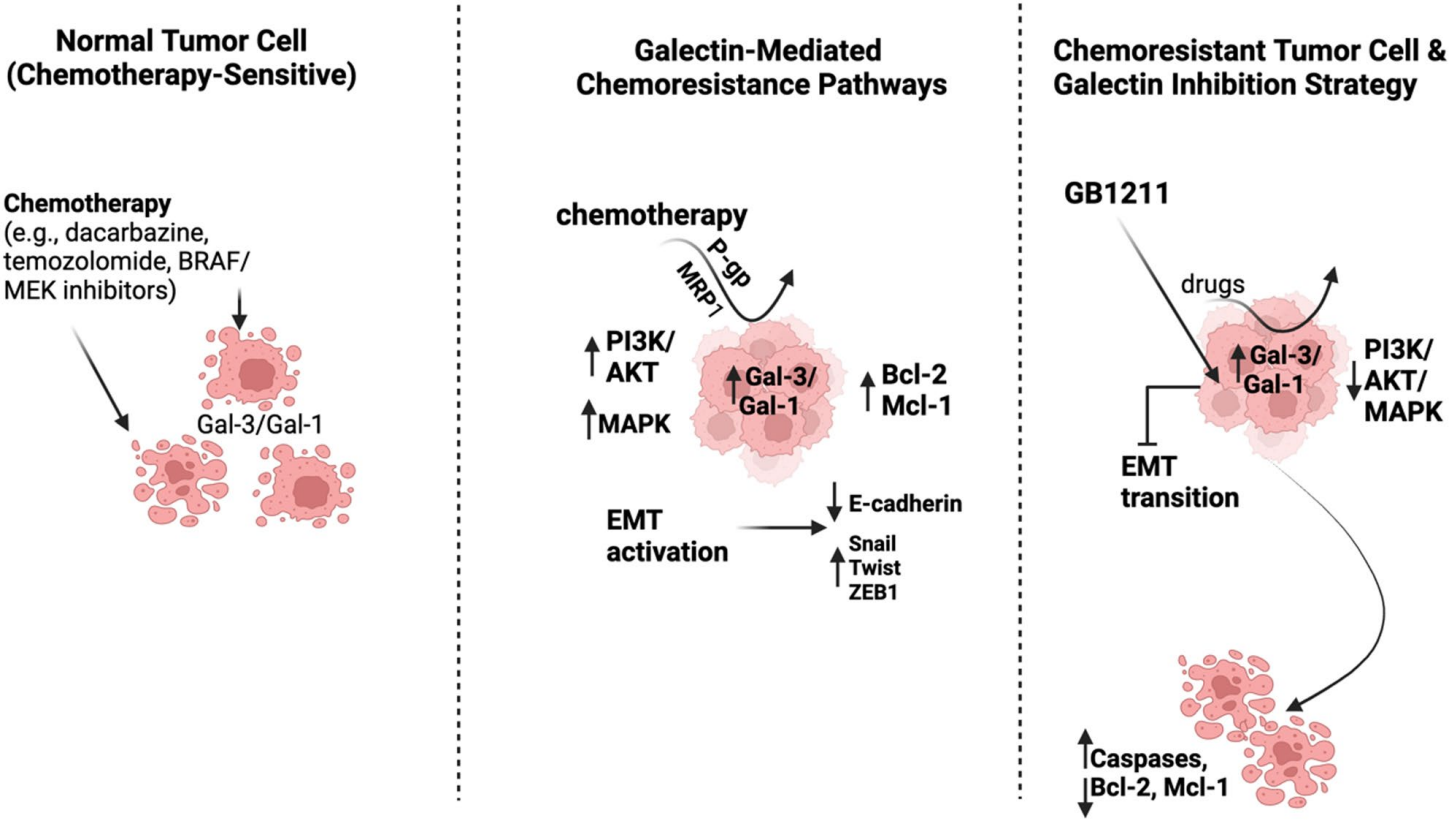
Galectin-mediated chemoresistance in melanoma and SCC. This figure illustrates how Gal-3 and Gal-1 contribute to chemoresistance skin cancers by promoting drug efflux, pro-survival signaling, and EMT. It also demonstrates how targeting galectins with specific inhibitors may restore chemosensitivity, offering a promising therapeutic approach in overcoming drug resistance. Created with Biorender.com

Given the role of galectins in chemoresistance, targeting them may improve response rates to standard therapies. Intracellular Gal-3 plays a crucial role in melanoma progression, as its downregulation in metastatic melanoma promotes migration, invasion, and metastasis by activating oncogenic pathways and upregulating the prometastatic transcription factor NFAT1, highlighting its potential as a therapeutic target [139].The combination of galectin inhibitors with traditional chemotherapy could therefore provide a promising strategy to combat resistance (Fig. 3).

### Radiotherapy resistance: galectins and DNA repair mechanisms

Radiotherapy is widely used for non-melanoma skin cancers and is also employed as adjuvant therapy in metastatic melanoma [140, 141]. However, radioresistance remains a significant barrier to treatment efficacy, and galectins contribute to this phenomenon by influencing DNA damage repair, cell survival, and tumor hypoxia adaptation [124].

Gal-3 plays a crucial role in DNA damage repair by interacting with BARD1, influencing homologous recombination, and affecting the G2/M checkpoint response, as demonstrated through GAL-3 silencing and protein interaction network analysis [142]. By enhancing these repair pathways, galectins promote tumor cell survival following radiotherapy, thereby reducing treatment efficacy.

Furthermore, Gal-1 is highly expressed in hypoxic tumor regions, where it plays a radioprotective role by activating HIF-1α, a key regulator of hypoxia-induced survival pathways [143]. Hypoxia is a well-established factor in radiotherapy resistance, as it reduces ROS generation, which is essential for radiation-induced tumor cell killing [144].

Additionally, post-radiotherapy immune activation is often counteracted by galectin-mediated immunosuppression [69]. Radiotherapy can induce an inflammatory response that recruits T cells to the tumor site; however, elevated levels of Gal-9 following radiation exposure have been linked to immune suppression by inducing apoptosis of activated T cells via TIM-3 signaling [12]. This suggests that targeting galectins could enhance the immunogenic effects of radiotherapy.

Recent research has explored galectin inhibitors as radiosensitizers, with promising preclinical results [145]. Inhibition of Gal-3 in melanoma models has been shown to impair DNA repair mechanisms, leading to increased tumor cell apoptosis following radiation exposure [13]. These findings support the potential for combination therapies that integrate galectin-targeting agents with radiotherapy to improve treatment outcomes.

## Challenges and future directions

Although significant progress has been made in understanding the role of galectins in skin cancers, several contradictory results and methodological limitations must be considered. Several contradictory findings regarding galectin functions in skin cancers must be acknowledged. For instance, Galectin-1 has been consistently linked to angiogenesis and immune evasion, yet its prognostic value varies depending on the tumor subtype and detection method [104, 146]. Similarly, while Galectin-3 is often associated with tumor progression, in certain epithelial contexts it may act as a differentiation factor and limit invasion [78, 147]. Galectin-7 provides another example of dual behavior, being implicated both in metastasis promotion and in pro-apoptotic functions [80, 148]. Finally, Galectin-9 illustrates the complexity of intracellular versus extracellular functions: it can induce immunosuppression via TIM-3 signaling, but has also been reported to enhance anti-tumor immunity and correlate with improved prognosis [11, 149]. These discrepancies highlight the context-dependent nature of galectin biology and underscore the need for standardized methodologies and well-defined patient cohorts to clarify their precise roles in skin cancer progression.

Although galectins have been extensively studied for their role in skin cancer metastasis, several challenges remain before they can be effectively used as therapeutic targets or biomarkers in clinical practice. Key areas of concern include tumor heterogeneity, biomarker development, and the integration of galectin-targeting strategies into precision medicine approaches.

One of the major challenges in targeting galectins is tumor heterogeneity, as galectin expression can vary significantly between different skin cancer subtypes and even among individual tumors within the same patient. Melanoma, SCC and BCC exhibit distinct levels and patterns of galectin expression, which directly influence their involvement in tumor progression and metastasis [91, 150]. Additionally, Gal-1, − 3 , and − 9 are differentially expressed in cervical cancer, with galectin-1 linked to poor survival and increased tumor invasion, galectin-3 showing variable effects on invasion, and galectin-9 potentially indicating improved survival, highlighting their potential as prognostic markers and therapeutic targets [151]. This variability presents a significant obstacle in developing galectin-targeted therapies, as compensatory mechanisms may allow tumor cells to upregulate alternative galectins, effectively bypassing the effects of single-galectin inhibitors and contributing to treatment resistance.

To address these challenges, advanced molecular profiling techniques such as single-cell RNA sequencing (scRNA-seq) and spatial transcriptomics can be utilized to characterize galectin expression heterogeneity at the individual tumor cell level [152]. These approaches enable a more precise understanding of galectin-driven tumor behavior, allowing for the identification of patient subgroups that may benefit from targeted interventions. Additionally, combination therapies that simultaneously target multiple galectins or integrate galectin inhibitors with immune checkpoint blockers (e.g., anti-PD-1, anti-CTLA-4) could provide a more effective treatment approach by preventing tumor adaptation and therapeutic resistance [130]. These strategies highlight the importance of personalized treatment paradigms, which take into account the tumor’s galectin expression profile to optimize therapeutic outcomes (Table 3).

**Table 3:** Challenges and potential solutions in galectin-targeting strategies

Challenge	Description	Potential solutions
Tumor heterogeneity	Variability in galectin expression among different skin cancer subtypes and even within the same tumor.	Single-cell sequencing to profile galectin heterogeneity; combination therapies targeting multiple galectins.
Biomarker validation	Lack of clinically validated diagnostic/prognostic markers using galectins.	Large-scale trials; non-invasive liquid biopsy approaches to monitor galectin levels.
Therapeutic targeting	Need for highly selective and effective galectin inhibitors.	Development of new galectin inhibitors with optimized specificity and minimal toxicity.
Integration into precision medicine	Personalized galectin-based therapies are not yet standard in clinical practice.	Stratification of patients based on galectin expression before treatment initiation.
Combination therapy uncertainty	The best combinations (galectin inhibitors + immunotherapy/chemotherapy) are not yet defined.	Preclinical studies and clinical trials to optimize combination strategies.

## Conclusions

In summary, galectins are central modulators of tumor–host interactions in skin cancers. By shaping immune evasion, stromal dynamics, and therapy resistance, they represent both a challenge and an opportunity for clinical intervention. Yet, their roles remain context-dependent and sometimes contradictory, highlighting the importance of standardized and integrative approaches. The development of selective galectin inhibitors, particularly in combination with immune checkpoint blockade, holds considerable promise. Ultimately, translating these insights into clinical practice will require bridging current gaps in knowledge and identifying reliable biomarkers for patient stratification.

## Data Availability

Not applicable.
